# Albumin-Stabilized Manganese Oxide/Semiconducting Polymer Nanocomposites for Photothermal-Chemodynamic Therapy of Hepatic Carcinoma

**DOI:** 10.3389/fbioe.2022.919235

**Published:** 2022-06-06

**Authors:** Qi Su, Changcun Liu, Jingyi Zhu, Mengbin Ding, Zhen Zhang, Jingchao Li, Qin Zhang

**Affiliations:** ^1^ Department of Critical Care Medicine, Shanghai General Hospital, Shanghai Jiao Tong University School of Medicine, Shanghai, China; ^2^ Department of Nuclear Medicine, Shanghai General Hospital, Shanghai Jiao Tong University School of Medicine, Shanghai, China; ^3^ School of Pharmaceutical Sciences, Nanjing Tech University, Nanjing, China; ^4^ Shanghai Engineering Research Center of Nano-Biomaterials and Regenerative Medicine, College of Chemistry, Chemical Engineering and Biotechnology, Donghua University, Shanghai, China; ^5^ Institute of Translational Medicine, Shanghai University, Shanghai, China

**Keywords:** hepatic carcinoma, photothermal therapy, chemodynamic therapy, combinational therapy, nanocomposites

## Abstract

Hepatic carcinoma is one of the most common cancers worldwide, while its treatment remains a great challenge. Traditional therapeutic methods often have disadvantages such as limited therapeutic efficacy and potential side effects. In this study, we report the construction of bovine serum albumin (BSA)–stabilized manganese oxide (MnO_2_)/semiconducting polymer (SP) nanocomposites to combine photothermal therapy (PTT) and chemodynamic therapy (CDT) for treatment of hepatic carcinoma in living mouse models. Such nanocomposites are composed of BSA, SP, and MnO_2_ as the stabilizer, PTT, and CDT agent, respectively. SP produced local heat under near-infrared (NIR) laser irradiation for PTT, and MnO_2_ nanoparticles mediated CDT in the tumor microenvironment, leading to apoptosis of cancer cells. Such nanocomposite-mediated combinational therapy showed a much higher efficacy in inhibiting growth of subcutaneous HepG2 tumors in nude mice than sole treatment. This study thus provides a multifunctional nanoplatform for safe and effective treatment of hepatic carcinoma.

## Introduction

Hepatic carcinoma has been the fifth common type of cancer and third common cause of cancer-related deaths over the world (Zhou et al., 2018; Liu et al., 2020; Qi et al., 2021). Traditional approaches for treatment of hepatic carcinoma include surgery, chemotherapy, and radiotherapy (Golubnitschaja et al., 2016; Wang et al., 2019; Zhu et al., 2019). Surgery is still one of the first-choice treatments for hepatic carcinoma, but tumor recurrence rates after surgery are higher than 40%, which restricts the long-term survival of patients (Depalo et al., 2017). Although chemotherapy has exhibited a remarkable curative effect with great success in clinical practice, it often has disadvantages such as poor tumor specificity, high systemic toxicity, and adverse effects (Tian et al., 2010). Radiotherapy usually faces the common dilemmas of poor efficacy, severe side effects, and radioresistance (De Ruysscher et al., 2019). Therefore, exploration of efficient and safe strategies for treatment of hepatic carcinoma is in high demand.

Unlike traditional treatment, photothermal therapy (PTT) utilizes photothermal conversion agents to convert light energy into heat energy, thereby increasing the temperature of the surrounding environment and causing the death of cancer cells (Hu et al., 2018; Jung et al., 2018; Liu et al., 2019). As external laser irradiation can precisely target tumor tissues, PTT can minimize the damage to the surrounding normal tissues and thus shows high specificity (Li et al., 2019; Li et al., 2020; Yuan et al., 2020). Chemodynamic therapy (CDT) is an emerging tumor treatment method that utilizes Fenton and Fenton-like reactions in the tumor microenvironment to generate highly toxic hydroxyl radicals (OH) for inducing cell apoptosis (Wang et al., 2020; Hao et al., 2021; Tian et al., 2021). CDT has high tumor specificity and selectivity and low toxicity in normal tissues, but the limited content of endogenous hydrogen peroxide (H_2_O_2_) and high concentrations of antioxidants in the tumor microenvironment can lead to unsatisfactory therapeutic effects (Cao et al., 2019; Ming et al., 2020; Yu et al., 2021). Since it is difficult for a single treatment method to achieve the desired therapeutic effect, a combination of different treatment modalities is expected to improve the antitumor efficacy (Li et al., 2018b; Hu et al., 2019; Chen et al., 2021). Therefore, the combination of PTT with CDT has not only shown high selectivity for cancer treatment but also improved therapeutic efficacy.

With the development of nanotechnology, a large number of nanoparticles with different functions and properties have been used as therapeutic agents for cancer treatment (Gai et al., 2018; Sahu et al., 2020; Siddique and Chow, 2020). Nanoparticles can improve the stability, water solubility, and pharmacokinetics of small-molecular anticancer drugs and allow the delivery of drugs into tumor sites for improved chemotherapy (Begines et al., 2020; Manzano and Vallet-Regí, 2020; Yu et al., 2020). Some nanoparticles can generate heat or highly toxic reactive oxygen species (ROS) upon external stimuli or reactions with endogenous chemical stimuli in the tumor microenvironment to show therapeutic actions (Chen et al., 2016; Son et al., 2020; Tang et al., 2020). In addition, nanoparticles can mediate combinational therapy through integrating different therapeutic components into a single nanosystem (Zhang et al., 2017; Zhang et al., 2018; Shrestha et al., 2019). Therefore, nanoparticle-mediated combinational therapy should provide an alternative strategy for treatment of hepatic carcinoma.

**FIGURE 1:**
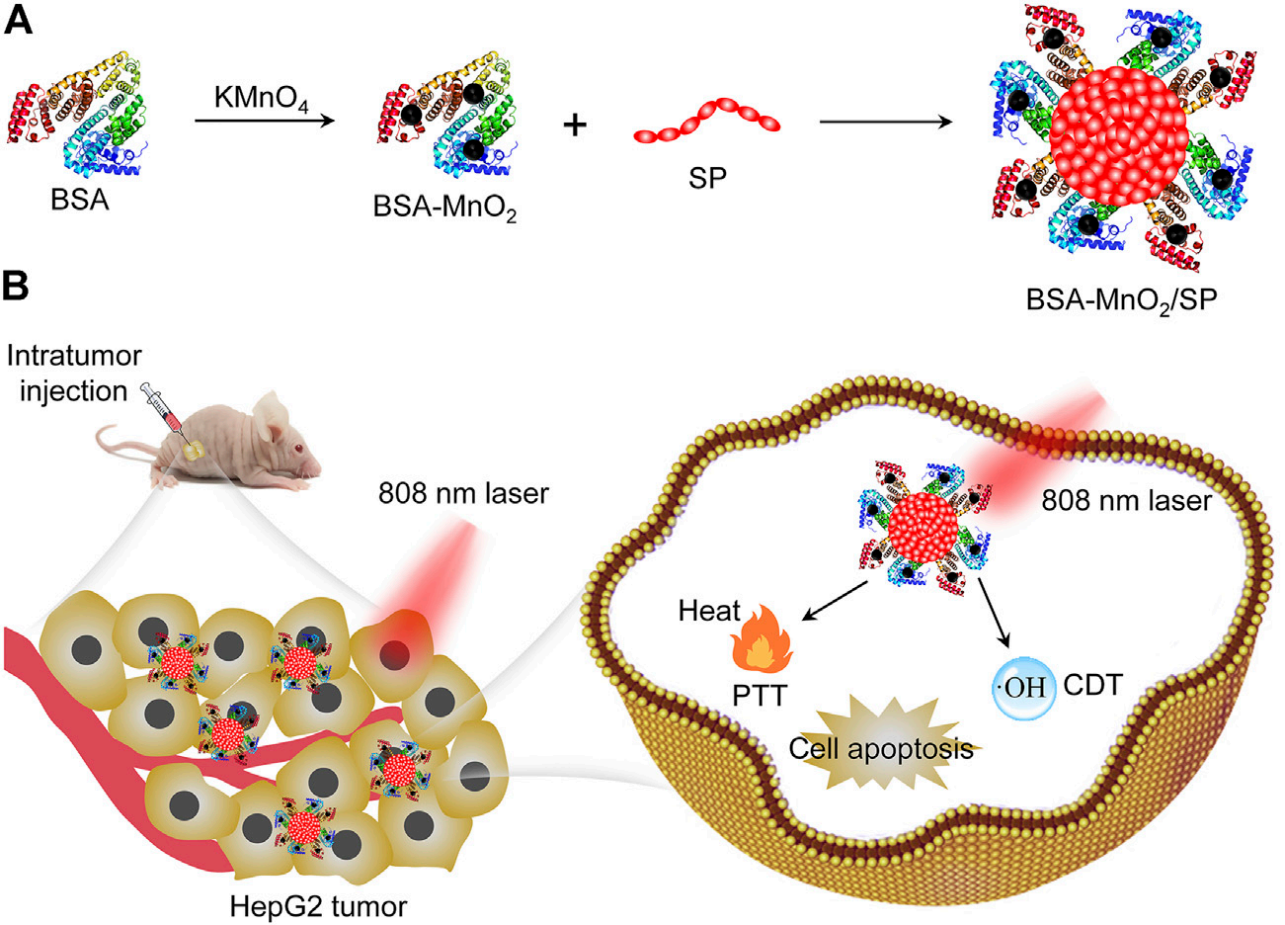
Construction of BSA-MnO_2_/SP nanocomposites for combinational PTT and CDT of hepatic carcinoma. **(A)** Schematic illustration of synthesis of BSA-MnO_2_/SP nanocomposites. **(B)** Schematic illustration of BSA-MnO_2_/SP nanocomposite–mediated combinational PTT and CDT.

Herein, we report the construction of bovine serum albumin (BSA)–stabilized manganese oxide (MnO_2_)/semiconducting polymer (SP) nanocomposites for combinational PTT and CDT of hepatic carcinoma in living mouse models. BSA was utilized as both a reductant and a template to synthesize BSA-MnO_2_ nanoparticles, which served as a stabilizer to construct BSA-MnO_2_/SP nanocomposites (Figure 1A). MnO_2_ nanoparticles have been utilized for cancer therapy as they can mediate Fenton-like reactions in the tumor microenvironment to generate OH (Ou et al., 2021). Due to its good biocompatibility and excellent optical property, SP can be used for PTT (Li et al., 2018a). Under 808 nm laser irradiation, MnO_2_ and SP mediated the generation of OH and local heat in tumors for CDT and PTT, respectively. Such a combinational therapy showed an improved efficacy in completely inhibiting the growth of subcutaneous HepG2 tumors in nude mice (Figure 1B).

## Materials and Methods

### Materials

BSA and SP were purchased from Sigma-Aldrich (St. Louis, United States). Singlet oxygen sensor green (SOSG) and the cell counting kit-8 (CCK-8) agent was purchased from Thermo Fisher Scientific (Invitrogen, United States) and Dojindo Laboratories (Kumamoto, Japan), respectively. RPMI 1640 medium, penicillin-streptomycin, and fetal bovine serum (FBS) were obtained from Gibco (Grand Island, NY, United States). Ultrapure water used in all experiments was prepared using a water purification system (PALL Cascada, MI, United States). All other chemicals were purchased from Sinopharm Chemical Reagent Co. Ltd. (Shanghai, China).

### Synthesis of BSA-MnO_2_/SP Nanocomposites

BSA-MnO_2_ nanoparticles were synthesized according to the procedures reported previously (Liu et al., 2021; Chen et al., 2022). In brief, aqueous solution of KMnO_4_ (3 ml, 10.5 mg/ml) was dropwise added to BSA solution (7 ml, 35.7 mg/ml) under sonication, and the resulting solution was vigorously stirred at 37°C for 2 h. After purification *via* dialysis (molecular weight cut-off = 8–14 kDa), BSA-MnO_2_ nanoparticles were obtained. To synthesize BSA-MnO_2_/SP nanocomposites, SP dissolved in tetrahydrofuran (1 ml, 0.5 mg/ml) was rapidly injected into the solution of BSA-MnO_2_ nanoparticles, and the resulting solution was treated by sonication for 5 min. After removal of tetrahydrofuran *via* a nitrogen flow and filtration of the remaining solution *via* a 220 nm PVDF filter, BSA-MnO_2_/SP nanocomposites were obtained. To synthesize BSA/SP nanoparticles as control, SP dissolved in tetrahydrofuran (1 ml, 0.5 mg/ml) was rapidly injected into BSA solution under sonication for 5 min.

### Characterization Techniques

Transmission electron microscope (TEM) images were obtained using a Tecnai G2 TEM (United States). Dynamic light scattering (DLS) and zeta potential measurement were conducted on a Zetasizer Nano-series (Nano-ZS90, Malvern, United Kindom). UV-vis spectra were obtained using a Persee spectrophotometer (TU-1810, Beijing, China). Fluorescence spectra were recorded on a fluorescence spectrophotometer (RF-6000, SHIMADZU, Japan).

### Evaluation of Photothermal Property

To evaluate the photothermal property of BSA/SP and BSA-MnO_2_/SP, the solutions containing BSA/SP or BSA-MnO_2_/SP at an SP concentration of 100 μg/ml were irradiated by 808 nm laser at different power densities (0.5, 1.0, and 1.5 W/cm^2^) for 5 min. Thermal images were obtained using a thermal infrared camera, and the temperatures during laser irradiation were recorded. In addition, the solutions of BSA/SP or BSA-MnO_2_/SP at different SP concentrations (12.5, 25, 50, and 100 μg/ml) were irradiated by 808 nm laser at a power density of 1.0 W/cm^2^ for 5 min to evaluate the influence of nanoparticle concentrations on temperature. Moreover, the solutions were treated by laser on/off at least five times to evaluate the photothermal stability of BSA/SP and BSA-MnO_2_/SP nanoparticles.

### Evaluation of OH Generation Efficacy

The solutions containing BSA-MnO_2_ or BSA-MnO_2_/SP at a Mn concentration of 10 μg/ml were mixed with the solution of methylene blue (MB) with or without the addition of glutathione (GSH, 0.5 mM) and H_2_O_2_ (100 µM). After incubation for 10 min, the absorbance of the solutions was measured using a Persee spectrophotometer (TU-1810, Beijing, China). To evaluate PTT-amplified OH generation, the mixed solutions were irradiated by 808 nm laser at a power density of 1.0 W/cm^2^ for 5 min, and then the absorbance of solutions was measured using a spectrophotometer.

### *In Vitro* Cytotoxicity Assay

HepG2 cancer cells were cultured in RPMI 1640 cell culture medium containing penicillin and streptomycin and 10% FBS at 37°C. The cells were seeded in 96-well plates (1 × 10^4^ cells/well) and cultured for 24 h, and then the cells were incubated with BSA/SP or BSA-MnO_2_/SP nanoparticles at different SP concentrations (6.25, 12.5, 25, 50, and 100 μg/ml) for 24 h. The cell culture medium was discarded, the cells were carefully washed with phosphate buffer solution (PBS), and a fresh medium containing a 10% CCK-8 agent was added into each well; then the cells were incubated for another 4 h. The absorbance of each well was measured using a microplate reader to calculate the cell viability. The cells treated with PBS were used as control.

### *In Vitro* Therapeutic Efficacy Evaluation

HepG2 cells seeded in 96-well plates (1 × 10^4^ cells/well) were incubated with BSA/SP or BSA-MnO_2_/SP nanoparticles at SP concentration of 100 μg/ml with or without the addition of H_2_O_2_ (100 μM) for 24 h. Then laser irradiation (808 nm, 1.0 W/cm^2^) of cells was conducted for 5 min, and the cells were incubated for another 12 h. After that, a CCK-8 assay was used to evaluate the cell viability.

### Establishment of HepG2 Tumor Models

Nude mice (female, 4–6 weeks) were purchased from JieSiJie Laboratory Animal Co. Ltd. (Shanghai, China). All animal experiments were conducted according to the procedures permitted by the Institutional Anima Care and Treatment Committee of Donghua University*.* The suspension of HepG2 cells in PBS was subcutaneously injected into the right flank of each mouse (2 × 10^6^ cells/mouse). HepG2 tumor–bearing nude mice were used for *in vivo* experiments after 10 days of tumor implantation.

### *In Vivo* PTT of Tumors

HepG2 tumor–bearing mice were randomly divided into two groups (*n* = 3), and PBS solutions of BSA/SP or BSA-MnO_2_/SP nanoparticles (20 μL, SP concentration = 250 μg/ml) were intratumorally administrated into tumor tissues. At 30 min after injection, the tumors were irradiated by 808 nm laser (1.0 W/cm^2^) for 10 min. During laser irradiation, a thermal camera was used to obtain thermal images of the mice, and the temperatures in the tumor sites were measured by analyzing the thermal images. Temperature curves as a function of laser irradiating time were obtained.

### *In Vivo* Antitumor Efficacy Evaluation

The HepG2 tumor–bearing mice were randomly divided into four groups: PBS, BSA-MnO_2_/SP, BSA/SP + laser, and BSA-MnO_2_/SP + laser (*n* = 4). The mice were intratumorally administrated with 20 μl PBS and PBS solutions of BSA/SP or BSA-MnO_2_/SP nanoparticles (SP concentration = 250 μg/ml). For laser irradiation groups, the tumors were irradiated by 808 nm laser (1.0 W/cm^2^) for 10 min at 30 min after injection. After treatment, the sizes of the tumors and the body weights of mice were recorded every 2 days for 18 days. The volumes of the tumors were calculated as follows: V = (length) × (width)^2^/2. The relative tumor volumes were calculated as V/V_0_ (V_0_ is the tumor volume at day 0). At day 18, the mice were sacrificed to extract tumors, and the tumor weights were measured.

### Statistical Analysis

The data were expressed as mean ± standard deviation. The statistically significant differences were analyzed *via* unpaired student’s *t*-test. *p < 0.05, **p < 0.01, and ***p < 0.001 denoted statistical significance.

## Results and Discussion

### Preparation and Characterization of BSA-MnO_2_/SP Nanocomposites

**FIGURE 2:**
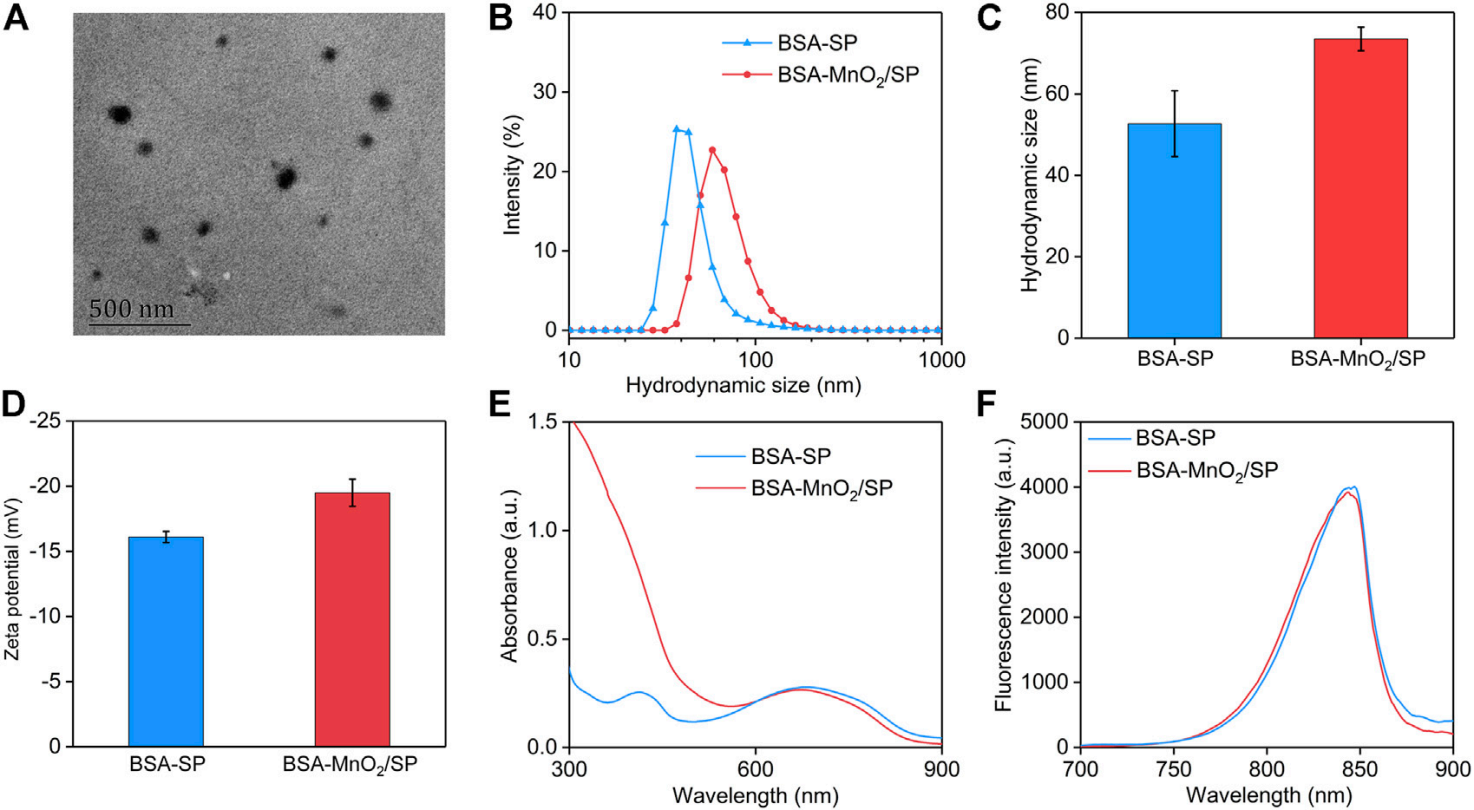
Preparation and characterization of BSA-MnO_2_/SP nanocomposites. **(A)** Representative TEM image of BSA-MnO_2_/SP nanocomposites. **(B)** DLS profiles of BSA-SP nanoparticles and BSA-MnO_2_/SP nanocomposites. **(C)** Hydrodynamic sizes of BSA-SP nanoparticles and BSA-MnO_2_/SP nanocomposites measured using DLS. **(D)** Zeta potentials of BSA-SP nanoparticles and BSA-MnO_2_/SP nanocomposites. **(E)** Absorbance spectra of BSA-SP nanoparticles and BSA-MnO_2_/SP nanocomposites. **(F)** Fluorescence spectra of BSA-SP nanoparticles and BSA-MnO_2_/SP nanocomposites.

BSA-MnO_2_ nanoparticles were first synthesized with BSA acting as both a reductant and a template. TEM imaging showed that the formed BSA-MnO_2_ nanoparticles had a spherical morphology (Supplementary Figure S1). The hydrodynamic size and zeta potential of BSA-MnO_2_ nanoparticles were measured to be 7.2 nm and −15.6 mV, respectively (Supplementary Figure S2). BSA-MnO_2_ nanoparticles were then used as a stabilizer to prepare BSA-MnO_2_/SP nanocomposites through sonication. Poly(cyclopentadithiophene-alt-benzothiadiazole) with excellent optical property and photothermal conversion efficacy was used as the SP (Li et al., 2018b). The formed BSA-MnO_2_/SP nanocomposites showed a spherical morphology, as observed from the TEM image (Figure 2A). The hydrodynamic size of BSA-MnO_2_/SP nanocomposites measured by DLS was 73.5 nm, which was larger than that of BSA-SP nanoparticles (52.7 nm) (Figures 2B,C). The zeta potential of BSA-MnO_2_/SP nanocomposites was measured to be −19.5 mV, which was similar to that of BSA-SP nanoparticles (−16.1 mV) (Figure 2D).

The optical properties of nanoparticles are important for their different applications (Peng and Chen, 2018; Peng et al., 2020a; Peng et al., 2020b), which were then investigated. The characteristic absorbance of SP at 680 nm was observed in the absorbance spectra of both BSA-SP and BSA-MnO_2_/SP (Figure 2E). In addition, BSA-SP nanoparticles and BSA-MnO_2_/SP nanocomposites had similar fluorescence property, with the fluorescence emission at around 845 nm (Figure 2F). These results indicating that the existence of MnO_2_ nanoparticles did not obviously affect the optical properties of nanoparticles.

### Evaluation of Photothermal Conversion Efficacy

**FIGURE 3:**
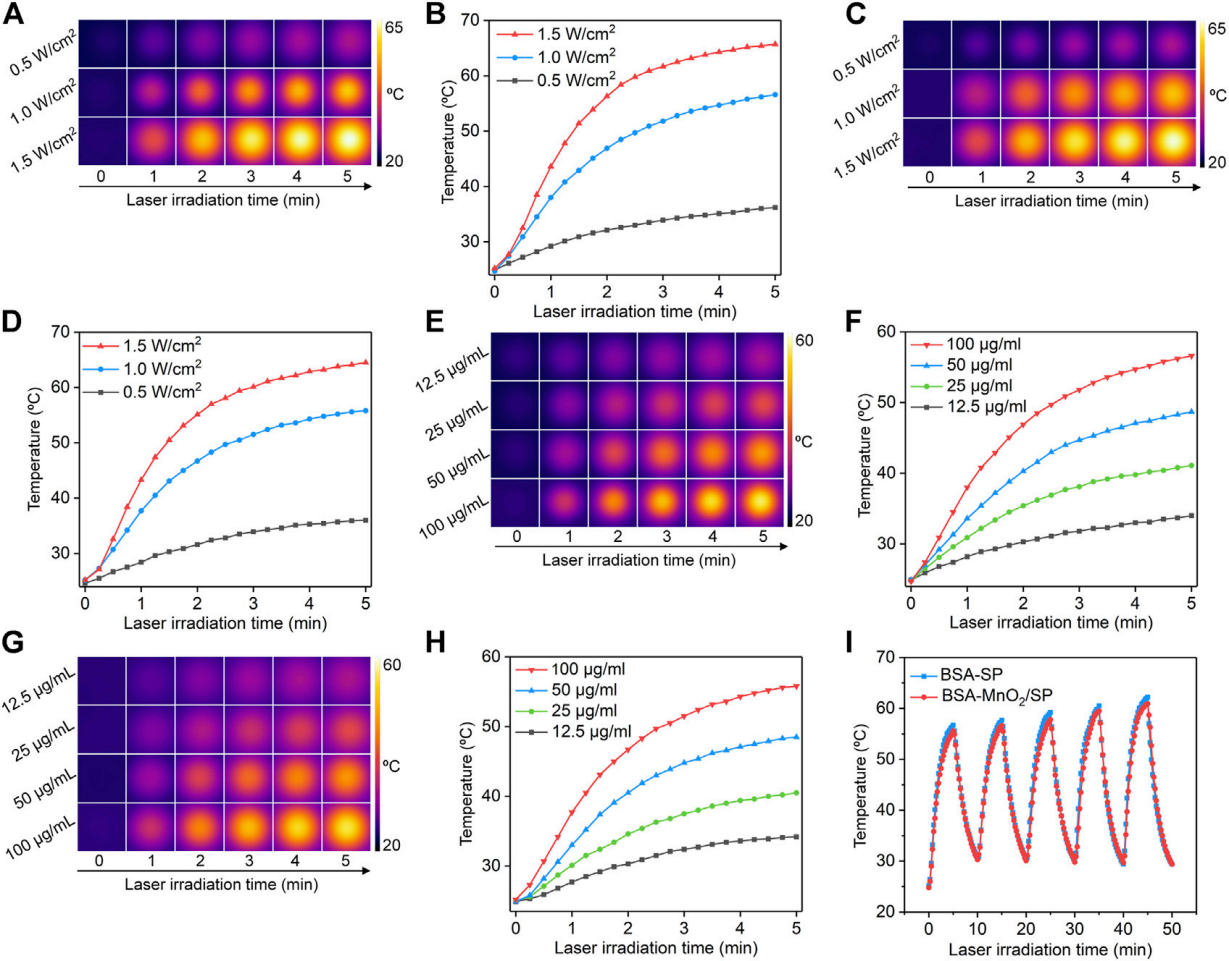
Evaluation of photothermal conversion efficacy. **(A)** Thermal imaging of solutions containing BSA-MnO_2_/SP nanocomposites under 808 nm laser irradiation at power densities of 0.5, 1.0, and 1.5 W/cm^2^. **(B)** Temperature curves of BSA-MnO_2_/SP nanocomposite solution under 808 nm laser irradiation at different power densities. **(C)** Thermal imaging of solutions containing BSA/SP nanoparticles under 808 nm laser irradiation at power densities of 0.5, 1.0, and 1.5 W/cm^2^. **(D)** Temperature curves of BSA/SP nanoparticle solution under 808 nm laser irradiation at different power densities. **(E)** Thermal imaging of solutions containing BSA-MnO_2_/SP nanocomposites at concentrations of 12.5, 25, 50, and 100 μg/ml under 808 nm laser irradiation at a power density of 1.0 W/cm^2^. **(F)** Temperature curves of BSA-MnO_2_/SP nanocomposite solutions at different concentrations under 808 nm laser irradiation (1.0 W/cm^2^). **(G)** Thermal imaging of solutions containing BSA/SP nanoparticles at concentrations of 12.5, 25, 50, and 100 μg/ml under 808 nm laser irradiation at a power density of 1.0 W/cm^2^. **(H)** Temperature curves of BSA/SP nanoparticle solutions at different concentrations under 808 nm laser irradiation (1.0 W/cm^2^). **(I)** Photothermal stability evaluation of BSA/SP nanoparticles and BSA-MnO_2_/SP nanocomposites after five cycles of laser on/off.

To evaluate the photothermal conversion efficacy, the solutions containing nanoparticles were treated with 808 nm laser, and the temperatures of the solutions were recorded. At the same concentration, the temperatures of the solutions containing BSA-MnO_2_/SP nanocomposites gradually increased under laser irradiation (Figure 3A). To confirm power density–dependent temperature increase, the commonly used laser densities (0.5, 1.0, and 1.5 W/cm^2^) were used. The temperature increased much more obviously at a higher power density of laser, which reached 36.2, 56.6, and 65.7°C after 5 min of laser irradiation at power densities of 0.5, 1.0, and 1.5 W/cm^2^, respectively (Figure 3B). After 5 min of laser irradiation, the temperatures of the solutions reached the maximum. These results suggested that higher power density could lead to a higher temperature increase for BSA-MnO_2_/SP nanocomposites. The power density–dependent temperature increase was also observed for BSA/SP nanoparticles (Figures 3C,D). At a power density of 1.0 W/cm^2^, the temperature of the solutions containing BSA-MnO_2_/SP nanocomposites increased diversely at different concentrations (Figure 3E). At concentrations of 12.5, 25, 50, and 100 μg/ml, the solution temperature increased to 34.0, 41.1, 48.7, and 56.6°C after 5 min of laser irradiation, respectively (Figure 3F). Similar tendencies were also observed for BSA/SP nanoparticles, and the temperature increase was much more obvious at a higher concentration (Figures 3G,H).

The photothermal stability of nanoparticles was then evaluated. After five cycles of laser on/off, the temperature increases of solutions containing BSA/SP nanoparticles and BSA-MnO_2_/SP nanocomposites did not have any decreases (Figure 3I), suggesting their excellent photothermal stability. The slight increases in maximal temperatures for solutions after more cycles of laser irradiation might be due to high temperature–mediated evaporation of water to increase the concentrations of nanoparticles. In addition, the temperatures were similar for BSA/SP nanoparticles and BSA-MnO_2_/SP nanocomposites at the same concentration and laser irradiation time. This indicated that BSA/SP nanoparticles and BSA-MnO_2_/SP nanocomposites had similar photothermal conversion efficacy.

### Evaluation of OH Generation Efficacy

**FIGURE 4:**
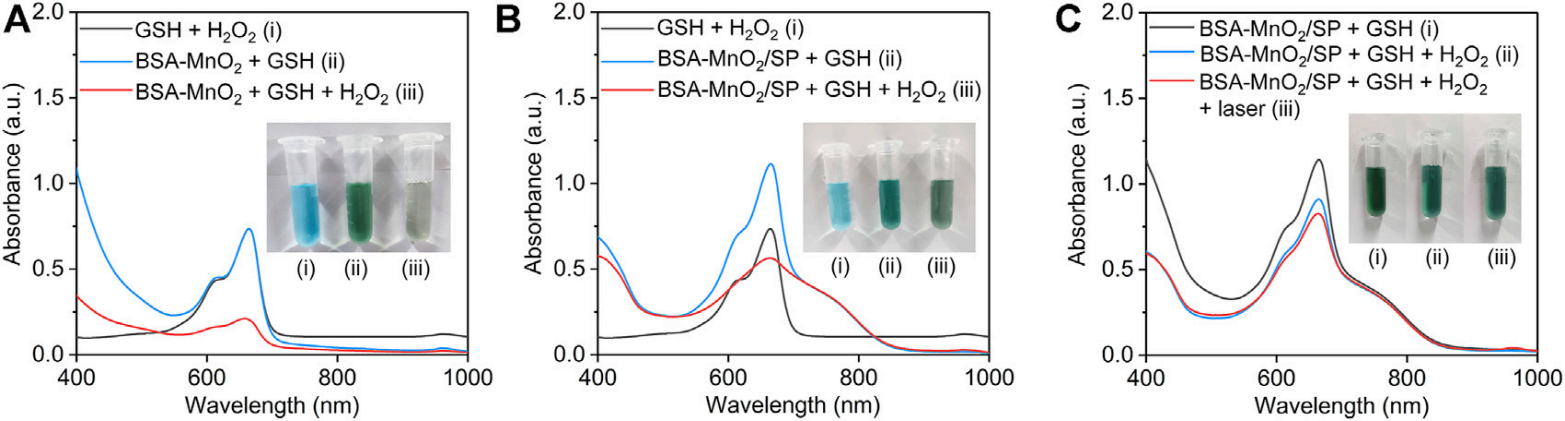
Evaluation of OH generation efficacy. **(A)** Evaluation of OH generation *via* BSA-MnO_2_ nanoparticle–mediated Fenton-like reaction using MB as an indicator. **(B)** Evaluation of OH generation *via* BSA-MnO_2_/SP nanocomposite–mediated Fenton-like reaction using MB as an indicator. **(C)** Evaluation of OH generation for BSA-MnO_2_/SP nanocomposites without or with 808 nm laser irradiation (1.0 W/cm^2^) for 5 min.

The generation of OH *via* MnO_2_-mediated Fenton-like reaction was evaluated using MB as the indicator. The characteristic absorption peak of MB at 665 nm did not have obvious changes for GSH + MB + H_2_O_2_ and BSA-MnO_2_ + GSH + MB groups, while that was obviously reduced for the BSA-MnO_2_ + GSH + MB + H_2_O_2_ group (Figure 4A), which suggested the generation of OH *via* Fenton-like reaction for BSA-MnO_2_ nanoparticles. The characteristic absorption peak of MB was still high in the BSA-MnO_2_/SP + GSH + MB group, which was obviously reduced in the BSA-MnO_2_/SP + GSH + MB + H_2_O_2_ group (Figure 4B), indicating the OH generation for BSA-MnO_2_/SP nanocomposites. In addition, the decrease in the characteristic absorption peak of MB in the BSA-MnO_2_/SP + GSH + MB + H_2_O_2_ group was much more obvious after 808 nm laser irradiation (Figure 4C), which suggested the enhanced generation of OH after 808 nm laser irradiation. These results suggested that the Fenton-like reaction effect of BSA-MnO_2_/SP nanocomposites could be accelerated by the PTT-mediated temperature rise, and thus, PTT and CDT showed a synergistic effect.

### Evaluation of *In Vitro* Therapeutic Efficacy

**FIGURE 5:**
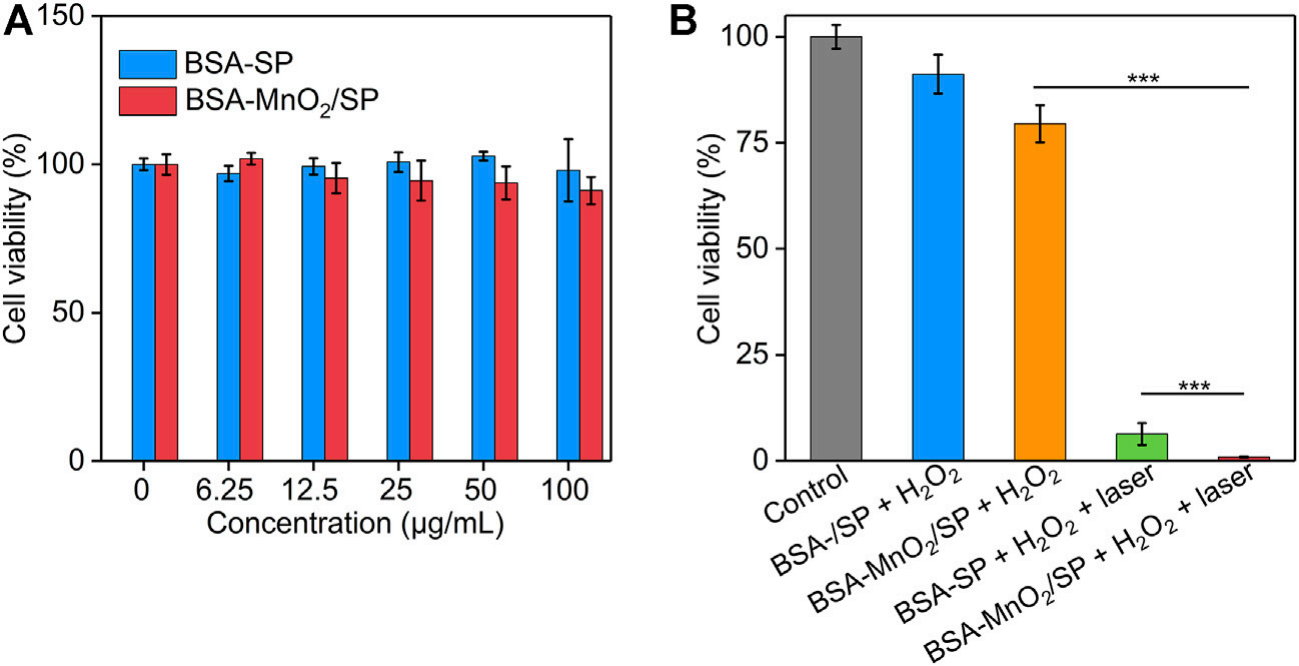
Evaluation of *in vitro* therapeutic efficacy. **(A)** Cell viability of HepG2 cells after incubation with BSA/SP nanoparticles or BSA-MnO_2_/SP nanocomposites at different SP concentrations for 24 h. **(B)** Cell viability of HepG2 cells after incubation with BSA/SP nanoparticles or BSA-MnO_2_/SP nanocomposites in the presence of H_2_O_2_ (100 μM) without or with 808 nm laser irradiation (1.0 W/cm^2^) for 5 min.

The *in vitro* cytotoxicity of BSA/SP nanoparticles and BSA-MnO_2_/SP nanocomposites was evaluated using the CCK-8 assay. After treatment with BSA/SP nanoparticles for 24 h, the cell viability of HepG2 cells did not have obvious changes even at a high concentration of 100 μg/ml when compared to the control group (Figure 5A), which indicated the good cytocompatibility of BSA/SP nanoparticles. After incubation with BSA-MnO_2_/SP nanocomposites, the cell viability reduced with the increase in concentration, which should be due to the slight cytotoxicity induced by MnO_2_ nanoparticles.

The *in vitro* therapeutic efficacy of nanoparticles was then investigated (Figure 5B). Without 808 nm laser irradiation, the cell viability of HepG2 cells did not have obvious changes after treatment with BSA/SP nanoparticles and H_2_O_2_, while that significantly reduced to 79.5% after treatment with BSA-MnO_2_/SP nanocomposites and H_2_O_2_, suggesting the generation of highly toxic OH for BSA-MnO_2_/SP nanocomposite–mediated CDT. With 808 nm laser irradiation, the cell viability of HepG2 cells after treatment with BSA/SP nanoparticles and H_2_O_2_ was only 6.3% due to BSA/SP nanoparticle–mediated PTT. In addition, the cell viability of HepG2 cells after treatment of BSA-MnO_2_/SP nanocomposites and H_2_O_2_ with 808 nm laser irradiation was 0.87%, which was 91.3- and 7.2-fold lower than that in the BSA-MnO_2_/SP + H_2_O_2_ and BSA/SP + H_2_O_2_ + laser group, respectively. These results indicated the amplified *in vitro* therapeutic efficacy of BSA-MnO_2_/SP nanocomposite–mediated combinational PTT and CDT compared to sole CDT and PTT.

### Evaluation of *In Vivo* Antitumor Efficacy

**FIGURE 6:**
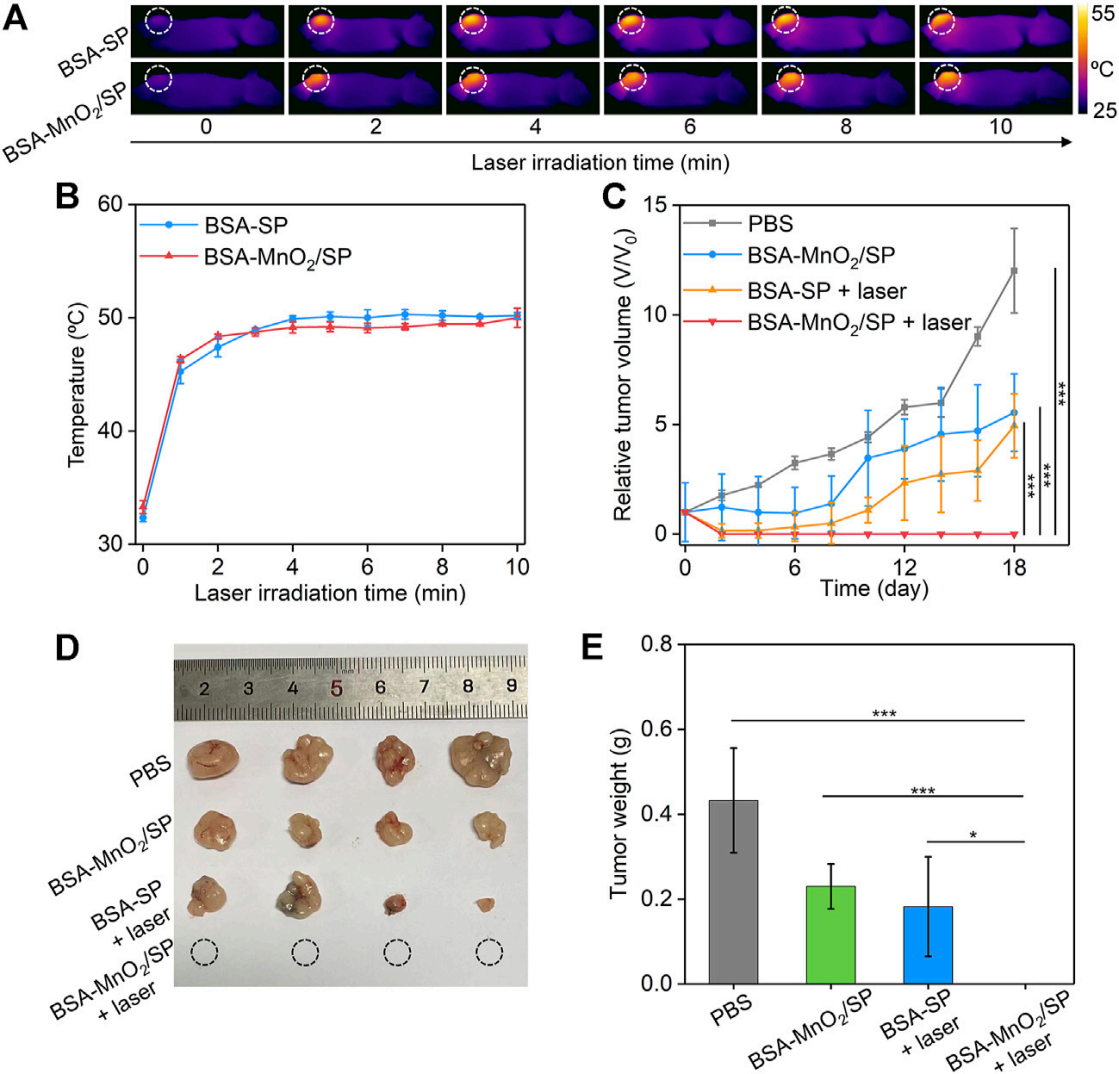
Evaluation of *in vivo* antitumor efficacy. **(A)** Thermal imaging of HepG2 tumor–bearing nude mice after treatment with BSA/SP nanoparticles or BSA-MnO_2_/SP nanocomposites under 808 nm laser irradiation (1.0 W/cm^2^) for different times. **(B)** Temperature changes in tumor sites for BSA/SP nanoparticle– or BSA-MnO_2_/SP nanocomposite–injected mice at different laser irradiating times. **(C)** Relative tumor volumes of HepG2 tumor–bearing mice after different treatments. **(D)** Photographs of tumors from HepG2 tumor–bearing mice after different treatments for 18 days. **(E)** Tumor weight of HepG2 tumor–bearing mice after different treatments for 18 days.

To evaluate the *in vivo* therapeutic efficacy, HepG2 tumor–bearing mice were randomly divided into four groups, and PBS, BSA/SP nanoparticles, or BSA-MnO_2_/SP nanocomposites were locally injected into tumor sites, followed by irradiation of tumors with 808 nm laser (1.0 W/cm^2^). Although the temperatures of nanoparticle solutions reached the maximum after 5 min of laser irradiation, a longer period of laser irradiation (10 min) was conducted to achieve ideal therapeutic efficacy. Under laser irradiation, the temperatures of tumor sites gradually increased with the laser irradiating time for both BSA/SP– and BSA-MnO_2_/SP–injected mice (Figure 6A). The temperatures at different irradiating times for these two groups were similar, which reached the maximum (around 50.2°C) after 10 min of laser irradiation (Figure 6B). Note that the temperature of tumor sites for PBS-injected mice did not have obvious changes under laser irradiation (Supplementary Figure S3). These results suggested that BSA/SP and BSA-MnO_2_/SP showed a similar *in vivo* PTT effect.

The *in vivo* therapeutic efficacy was evaluated by monitoring tumor growth. The tumor growth of BSA-MnO_2_/SP–injected mice without laser irradiation was inhibited by 2.2-fold compared to that in control mice (Figure 6C), which should be due to the CDT effect. The tumor growth was inhibited by 2.4-fold after BSA/SP nanoparticle injection with 808 nm laser irradiation because of the PTT effect. It should be noteworthy that the growth of tumors was completely inhibited after BSA-MnO_2_/SP nanocomposite injection and laser irradiation, which was due to the combinational action of PTT and CDT. After treatment for 18 days, no tumors were observed for the BSA-MnO_2_/SP + laser group, and the tumors in the BSA-MnO_2_/SP and BSA/SP + laser groups were smaller than those in the control group (Figure 6D). The tumor weight in the BSA-MnO_2_/SP + laser group was 0 g, while that was 0.43, 0.23, and 0.18 g for the control, BSA-MnO_2_/SP, and BSA/SP + laser group, respectively (Figure 6E). These results suggested that BSA-MnO_2_/SP after laser irradiation showed the highest antitumor efficacy due to the combinational action of CDT and PDT. In addition, the body weight of mice after different treatments remained nearly unchanged (Supplementary Figure S4), indicating that BSA-MnO_2_/SP–mediated therapy did not cause significant toxicity.

## Conclusion

We have reported the construction of BSA-MnO_2_/SP nanocomposites for combinational PTT and CDT of hepatic carcinoma in living mouse models. Such BSA-MnO_2_/SP nanocomposites could be synthesized *via* a facile two-step procedure. The formed BSA-MnO_2_/SP nanocomposites showed a high photothermal conversion efficacy under 808 nm laser irradiation and efficient OH generation efficacy *via* a Fenton-like reaction. By mediating the combinational action of PTT and CDT, BSA-MnO_2_/SP nanocomposites led to much higher efficacy in killing HepG2 cancer cells *in vitro* than to their counterparts. Such a treatment strategy could afford an obviously enhanced antitumor efficacy in inhibiting the growth of subcutaneous HepG2 tumors in living mice. In view of the flexible and facile construction of nanocomposites, this nanoplatform can be integrated with other therapeutic components (such as chemodrugs and immunotherapeutic drugs) to achieve multimodal therapy of different types of tumors. By modifying the targeting ligands on the surface to increase their accumulation into tumor sites after systematic administration, these nanocomposites can be used for targeted treatment of tumors.

## Data Availability

The raw data supporting the conclusion of this article will be made available by the authors, without undue reservation.
