# Supplementary material for: Albumin-Stabilized Manganese Oxide/Semiconducting Polymer Nanocomposites for Photothermal-Chemodynamic Therapy of Hepatic Carcinoma
Source: Front Bioeng Biotechnol. 2022 Jun 6;10:919235. doi: 10.3389/fbioe.2022.919235 (PMC9207483; doi:10.3389/fbioe.2022.919235)
Supplement: Supplementary file 1 [file Table1.DOCX]

Supplementary Material

Albumin-stabilized manganese oxide/semiconducting polymer nanocomposites for photothermal-chemodynamic therapy of hepatic carcinoma

Qi Su^1#^, Changcun Liu^2#^, Jingyi zhu^3*^, Mengbin Ding^4^, Zhen Zhang^4^, Jingchao Li^4*^ Qin Zhang^5^*

^1^Department of Critical Care Medicine, Shanghai General Hospital, Shanghai Jiao Tong University School of Medicine, Shanghai 201600, P.R. China

^2^Department of Nuclear Medicine, Shanghai General Hospital, Shanghai Jiao Tong University School of Medicine, Shanghai 201600, P. R. China

^3^School of Pharmaceutical Sciences, Nanjing Tech University, Nanjing 211816, P. R. China

^4^Shanghai Engineering Research Center of Nano-Biomaterials and Regenerative Medicine, College of Chemistry, Chemical Engineering and Biotechnology, Donghua University, Shanghai 201620, P.R. China. Email: jcli@dhu.edu.cn

^5^Institute of Translational Medicine, Shanghai University, Shanghai 200444, P. R. China. Email: sabrina_1985@shu.edu.cn

^#^Qi Su and Changcun Liu contributed equally to this work.


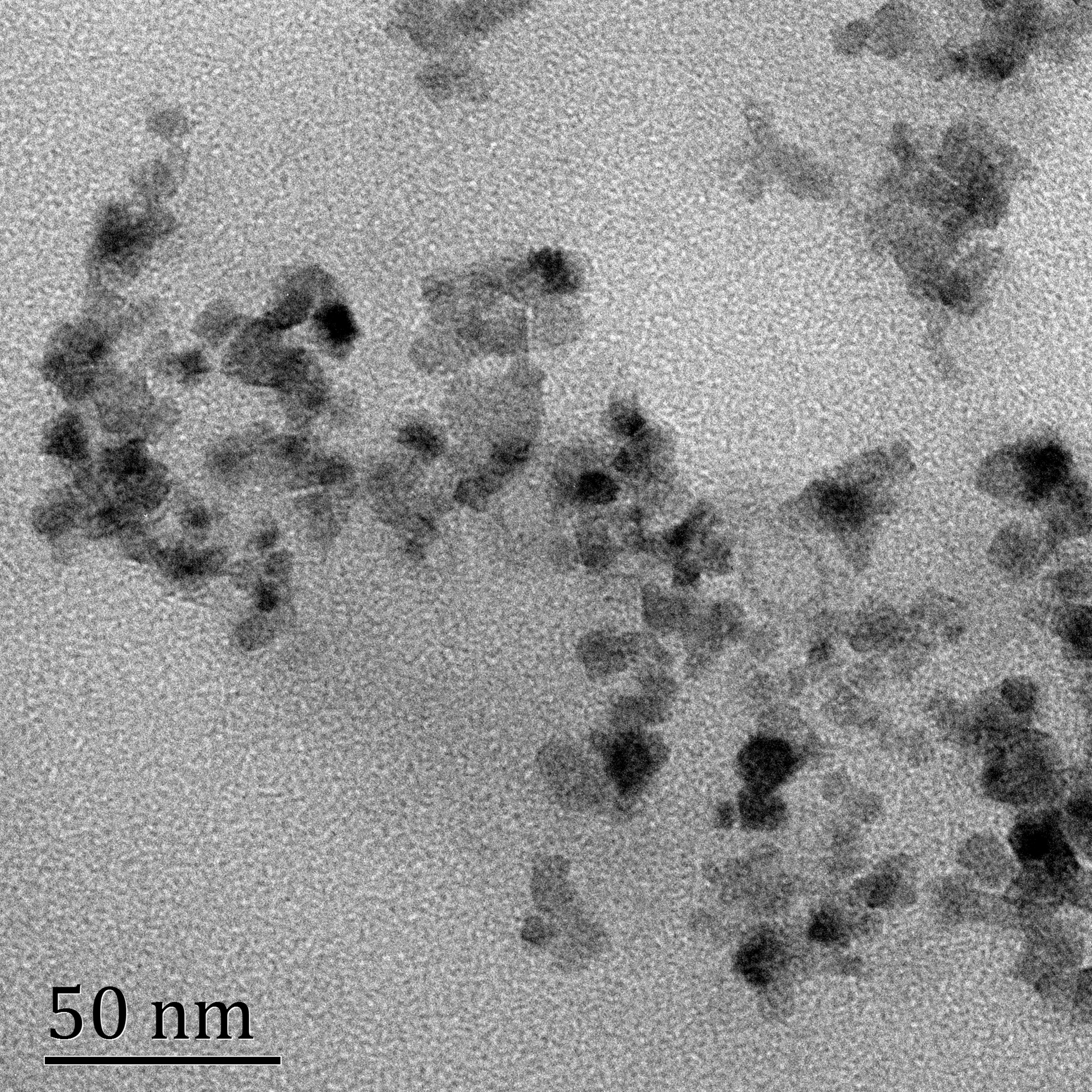


**Supplementary Figure 1.** Representative TEM image of BSA-MnO_2_ nanoparticles.


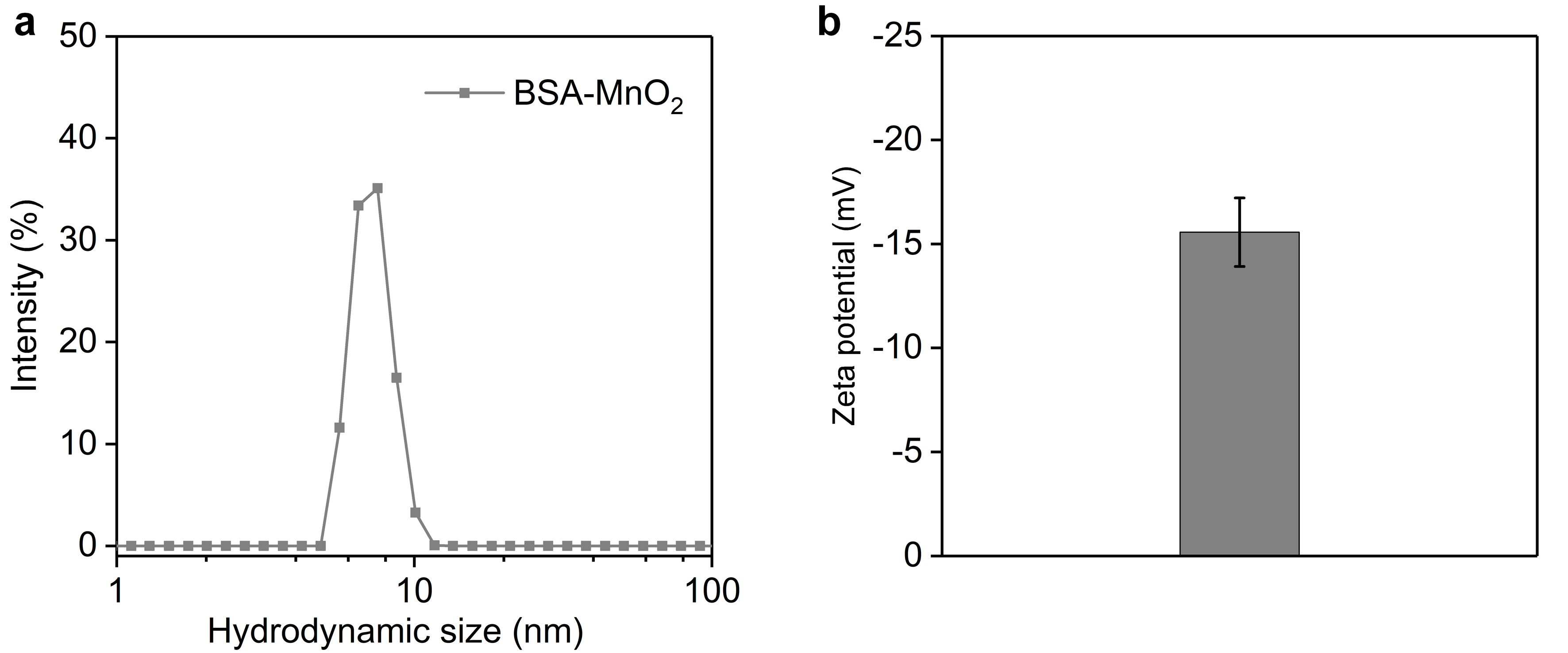


**Supplementary Figure 2.** Hydrodynamic size (a) and zeta potential (b) of BSA-MnO_2_ nanoparticles.


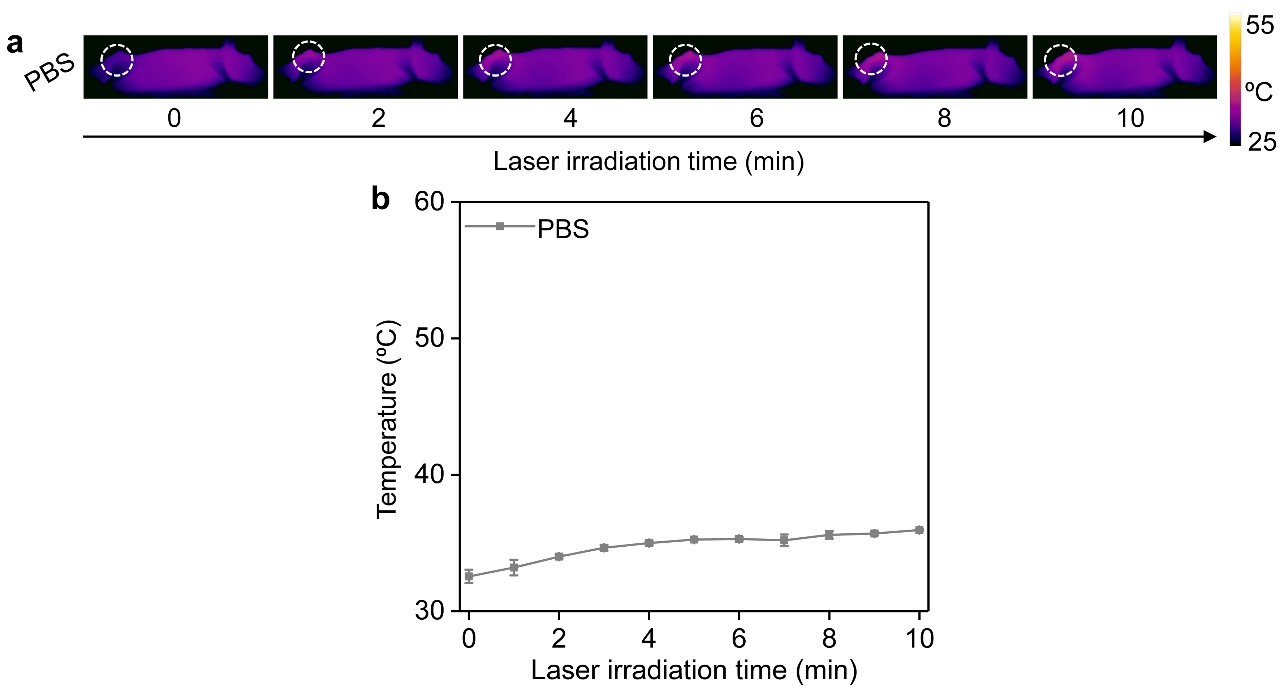


**Supplementary Figure 3.** (a) Thermal imaging of HepG2 tumor-bearing nude mice after PBS injection under 808 nm laser irradiation (1.0 W/cm^2^) for different time. (b) Temperature changes of tumor sites for PBS-injected mice at different laser irradiating time.

**
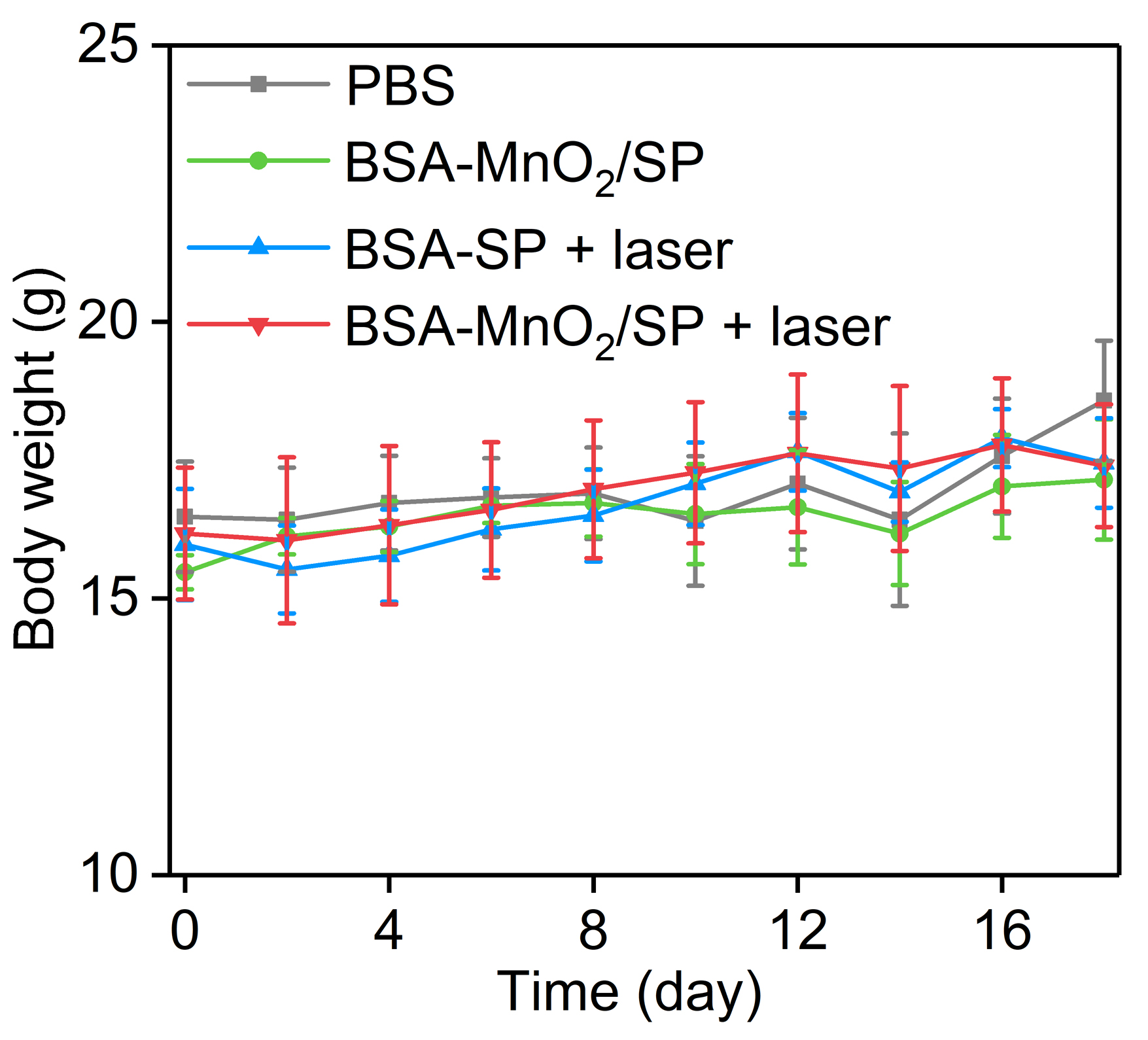
**

**Supplementary Figure 4.** Body weight of HepG2 tumor-bearing mice after different treatments.
